# Risk of falls in Brazilian elders with and without low back pain assessed using the Physiological Profile Assessment: BACE study

**DOI:** 10.1590/bjpt-rbf.2014.0183

**Published:** 2016-09-22

**Authors:** Nayza M. B. Rosa, Bárbara Z. Queiroz, Renata A. Lopes, Natalia R. Sampaio, Daniele S. Pereira, Leani S. M. Pereira

**Affiliations:** 1 Programa de Pós-graduação em Ciências da Reabilitação, Departamento de Fisioterapia, Universidade Federal de Minas Gerais (UFMG), Belo Horizonte, MG, Brazil

**Keywords:** low back pain, elders, risk of falls, falls, physiological profile assessment, physical therapy

## Abstract

**Background:**

Low back pain (LBP) is a common musculoskeletal condition among elders and is associated with falls. However, the underlying biological risk factors for falling among elders with LBP has been poorly investigated. The Physiological Profile Assessment (PPA) is a validated fall-risk assessment tool that involves the direct assessment of sensorimotor abilities and may contribute to the understanding of risk factors for falls among elders with LBP.

**Objective:**

To assess fall risk using the PPA in elders with and without LBP.

**Method:**

This is an observational, comparative, cross-sectional study with elders aged ≥65 years. The present study was conducted with a subsample of participants from the Back Complaints in the Elders (BACE) - Brazil study. Fall risk was assessed using the PPA, which contains five tests: visual contrast sensitivity, hand reaction time, quadriceps strength, lower limb proprioception, and postural sway.

**Results:**

Study participants included 104 individuals with average age of 72.3 (SD=4.0) years, divided into two groups: GI) 52 participants with LBP; GII) 52 participants without LBP. The participants with LBP had a significantly higher fall risk (1.10 95% CI 0.72 to 1.48), greater postural sway (49.78 95% CI 13.54 to 86.01), longer reaction time (58.95 95% CI 33.24 to 84.65), and lower quadriceps strength (–4.42 95% CI –8.24 to –0.59) compared to asymptomatic participants. There was no significant difference for vision and proprioception tests between LBP and non-LBP participants.

**Conclusion:**

Elders with LBP have greater risk for falls than those without LBP. Our results suggest fall-risk screening may be sensible in elders with LBP.

## BULLET POINTS

- Older people with low back pain (LBP) had a significantly higher fall risk as evaluated by the Physiological Profile Assessment.
- Greater postural sway was observed in elders with LBP.
- The LBP group had significantly lower quadriceps strength than the control group.
- Longer reaction time was found in older adults with LBP.
- Fall risk screening may be important in older people with LBP.

## Introduction

Falls are the third cause of disability among older people and a public health problem with great social impact worldwide in countries with a significant aging population^1^. Approximately 30% of Brazilian elders suffer from falls at least once a year, and almost half of them fall two or more times per year^2^. The main consequences of falls include fractures, increased dependency, institutionalization, as well as association with high rates of morbidity and mortality^1^. The assessment of fall risk in older adults is complex due to the multifactorial nature of underlying risk factors. Systematic reviews indicate that a multifactorial assessment of risk factors, followed by targeted intervention, is an effective strategy for preventing falls in this group^3^.

The Physiological Profile Assessment (PPA) is a validated fall-risk assessment tool that involves the direct assessment of sensorimotor abilities. PPA assesses vision, proprioception, muscle strength, reaction time, and postural sway^4^. Recently, our research group conducted an intra- and inter-rater reliability study of the PPA in a Brazilian elderly population^5^. The study findings indicated that the PPA composite score, and most component parts, had acceptable intra- and inter-rater reliability, and thus the PPA can be considered a reliable instrument for the assessment of fall risk in Brazilian older people^5^.

Low back pain (LBP) is a musculoskeletal condition most commonly found in those over 75 years, with a prevalence of 12 to 42% in subjects over 65 years^6^. A systematic review showed that the prevalence of severe back pain increases, while less severe pain decreases, with increasing age^7^. A systematic review on the prevalence of LBP in Brazil showed prevalence rates of 4.2% to 14.7% for LBP in the general population^8^.

Despite a high prevalence of LBP in elders, research is focused mainly on the economically active population, aged between 18 and 65 years. There are few studies with older people^9^. The prevalence of alterations present in senescence and senility, such as sarcopenia, osteoarthritis, osteoporosis, spinal stenosis, and other health conditions, makes the causes of LBP in elders specific to this age group. Indeed, LBP is associated with several adverse consequences in older people, including increased disability, number of falls, hospitalization, and institutionalization^9^.

Leveille et al.^10^ suggest that, because falls are of multifactorial origin, there is more than one mechanism by which musculoskeletal pain is associated with falls. Some mechanisms underlying the relationship between pain and falls that may interfere with worse balance control are neuromuscular effects of pain and changes in musculoskeletal systems.

In elders, pain can lead to muscle weakness or a slower neuromuscular response when trying to avoid an imminent fall^10^. When considering changes in musculoskeletal systems in older patients with LBP, changes inherent to senescence of the musculoskeletal system can be present, and one of the most common is osteoarthritis.

It is also important to consider sarcopenia, a common phenomenon of aging^11^. Sarcopenia may be associated with negative outcomes, such as disability, weakness of the stabilizing muscles of the spine, decreased mobility, and postural changes overloading the spine. Such modifications may increase the risk of falls in older adults. In addition to the changes that occur with aging, muscle and sensory changes that accompany LBP can contribute to balance changes, and therefore, to falls^10^ ^,^ ^12^.

Considering the gap in the literature regarding investigation of LBP in older people and the association of this disorder with falls, the aim of the present study was to evaluate the risk of falls using the PPA in two elderly groups: with LBP (GI) and without LBP (GII).

## Method

### Study design and participants

The Back Complaints in the Elders (BACE) consortium is a prospective cohort study^9^. The subsample of convenience of elders who participated in the GI consisted of participants from the BACE-Brazil (BACE B) study. This is an observational, comparative, cross-sectional study with people aged 65 and over who had a new episode of LBP. LBP was defined as pain in the area between the shoulder blades and the S1 vertebrae^13^. The episode was defined as new if the person did not seek for care due to LBP during the six months before data collection. For the BACE B study, participants would also have to present with an exacerbation of symptoms, which was defined as an episode of acute pain within six weeks of the recruitment period. An episode of LBP was defined as a period of pain in the lower back lasting for more than 24 hours, preceded and followed by a period of at least 1 month without LBP^14^.

For the BACE B study, older adults with LBP complaints were recruited by convenience. Firstly, they were referred to the BACE B research team by physicians or allied health care professionals from either public or private healthcare services in the city. Then, they were screened by the research team to see if they could be included in the study, according to the previously stated criteria. All subjects were clinically stable and fully capable of walking by themselves with or without walking aids.

The GII group included older adults, aged ≥65 years, without LBP. All subjects were clinically stable and fully capable of walking with or without walking aids. The sample of GII was recruited from elderly groups or from the waiting list of Escola de Educação Física, Fisioterapia e Terapia Ocupacional (School of Physical Education, Physical Therapy and Occupational Therapy) of Universidade Federal de Minas Gerais (UFMG), Belo Horizonte, MG, Brazil, after verification of the inclusion and exclusion criteria.

Participants were excluded from GI and GII if they presented any severe visual, motor, or hearing loss that would prevent them from being assessed during data collection. Individuals with the possibility of cognitive dysfunctions were excluded based on the scores of the Mini-Mental State Examination (MMSE) according to the level of education using the following cutoff points: 13 for illiterates, 18 for individuals with one to seven years education, and 26 for eight years or more of schooling^15^. Other exclusion criteria included disorders of the vestibular system, serious sequelae from stroke with localized loss of strength, neurological diseases and/or motor disabilities that would prevent participants from performing the functional tests, orthopedic surgeries to lower limbs (LL) in the last 3 months, amputation or recent history of fractures to the LL, or being in a wheelchair or bedridden.

The BACE Brazil study was approved by the Ethics Committee of UFMG (Approval number 0100.0.203.000-11). All participants signed an informed consent form.

Using the mean and standard deviation from a pilot study of 10 healthy elders, we calculated the effect size index values (d) for each variable. From these values, it was estimated that a sample size of 52 subjects in each group would be required in order to provide 80% power with a significance level set at 0.05.

### Measuring instruments

To characterize the sample population, participants answered an elaborate sociodemographic and clinical questionnaire that was standardized by the group of researchers involved in the BACE study^9^ and delivered as an interview by trained researchers.

**Fall Risk:** assessed with the PPA short form (Prince of Wales Medical Research Institute)^4^_._ The authors of the PPA identified the most important items for discriminating between fallers and non-fallers^16^ ^,^ ^17^. Based on a participant’s performance, the PPA computes a standardized fall risk score that has a 75% predictive accuracy for falls in the elders. The composite PPA score is derived from discriminant function analysis using data from large-scale studies^16^ ^,^ ^17^_._

The results of these tests are inputted into a software program (FallScreen^©^) and adjusted for age and sex. The program computes a fall risk ratio by using an algorithm. This test and its psychometric properties have been validated with good psychometric properties^16^_._ Global PPA scores indicate risk levels as follows: <0 low, 0-1 mild, 1-2 moderate, and >2 high fall risk.

### Visual contrast sensitivity

Visual contrast sensitivity was assessed using the Melbourne Edge Test^18^. The chart has 20 circular 25-mm-diameter patches containing edges with reducing contrast and with variable orientation as the identifying feature. The edges are presented in the following orientations: horizontal, vertical, 45 degrees to the left, and 45 degrees to the right. A card with the possible choices is presented to the participant. The lowest contrast patch identified correctly is recorded as the participants contrast sensitivity in decibel units, where 1 dB=10log^10^ contrast.

### Proprioception

Proprioception was assessed in the PPA using an established and validated lower limb-matching task. The participants are seated with their eyes closed and are asked to align their LL simultaneously on each side of an acrylic panel (60×60×1cm). The panel, marked with a protractor, is positioned between the participant’s legs. Any difference in aligning the LL is measured in degrees. After three practice trials, an average of five experimental trials is recorded^4^.

### Muscle strength

The maximum isometric muscle strength of the quadriceps was measured using a digital dynamometer attached to the participant’s dominant leg with a strap placed 10 cm above the ankle joint, and with the angles of the hip and knee at 90° with the participant seated^4^. The participant attempts to push against the strap. The best of three trials was recorded in kilograms^4^.

#### Reaction time

Reaction time was assessed in milliseconds using a handheld electronic timer with a light as a stimulus. The timer requires depression of a switch with a finger as the response. The timer has a built-in variable delay of 1 to 5 seconds to remove any cues. A modified computer mouse was used as the response box for the finger press task. Five practice trials were undertaken, followed by ten experimental trials^4^.

### Postural sway

Postural oscillation was measured using a sway meter that measures the body dislocation in the participant’s waist level, according to Lord et al.^4^. The equipment consists of a 40-cm rod with a vertically mounted pen at its end. The rod is strapped to the participant’s lower back with a belt so that it extends posteriorly. While the subject tries to stand as motionless as possible for 30 seconds, the pen records the oscillation on a millimeter graph paper attached to an adjustable-height table. The test was performed with the subject with eyes open standing on a foam rubber mat 15 cm high^17^. The anteroposterior and mediolateral oscillations are recorded.

**Falls:** they were evaluated using the following questions: “Did you fall in the last 12 months?” The participant should answer either yes or no and if yes, how many times they fell. “Where did you fall?” Participants must choose between the following answers: indoors or outdoors. “Why did you fall?” There were two possible answers: accidental or non-accidental. In addition, participants should answer yes or no to the following questions: “Did you sustain a fracture because of falling?” and “Were you hospitalized because of falling?”

Falls were defined as “events that resulted in a person coming to rest unintentionally on the ground or another lower level, not as the result of a major intrinsic event or an overwhelming hazard”^19^.

**LBP intensity:** during the time of assessment, it was evaluated using the Numerical Pain Scale (NPS). 0 indicated no pain, while 10 indicated the worst pain possible. This scale is simple and easy to implement and its use has been reported internationally in elders with high reliability and reproducibility^20^.

The short version of the Geriatric Depression Scale (GDS-15) was used to quantify depression symptoms^21^, and the International Physical Activity Questionnaire (IPAQ) was used to investigate the physical activity levels of participants^22^.

### Statistical analysis

Descriptive statistics were used for sample characterization. The Kolmogorov-Smirnov test was used to verify the distribution of the data. A comparison analysis between groups for continuous variables was performed by independent t-test for normally distributed data or the nonparametric Mann-Whitney U test for data with non-normal distributions. The Chi-square test was used for comparisons of categorical variables. All of the analyses involved a significance level of *α*=5% and confidence intervals of 95%, using the Statistical Package for the Social Sciences version 15.0.

There was no loss of any data assessed.

## Results

Study participants included 104 elders. Most of the sample consisted of divorced, separated, or widowed individuals. The most prevalent comorbidities were arterial hypertension and osteoarthritis. A description of the clinical and demographic variables is included in Table 1.

**Table 1:** Comparison between groups GI and GII: descriptive variables.

	**Low Back Pain Group (GI)** n=52		**Control Group** **(GII)** n=52			*p value*	
	Mean (SD)		Mean (SD)				
Age (years)	70.6 (3.9)		74.1 (4.2)			0.46^#^	
Education (years)	8.0 (4.1)		8.1 (3.9)			0.37^#^	
Number of medications	4.0 (1.8)		3.5 (1.3)			0.21^#^	
Number of comorbidities	2.4 (1.2)		1.7 (1.0)			0.00^*#^	
Level of physical activity (MET,min/week)	1879.3 (1831.4)		2270.9 (1841.9)			0.40^#^	
Depressive symptoms (GDS score/15)	4.9 (2.6)		2.6 (1.8)			0.00^*#^	
Psychotropic medications%							
Yes	21.2%		7.7%			0.05^†^	
Sex %							
Female	92.3%		88.5%			0.52^†^	
Living Alone %							
Yes	15.4%		26.9%			0.17^†^	
LBP intensity (NPS score /10)	4.1 (3.3)						

* Significant difference p<0.01.

# Mann-Whitney U test.

† Chi-square test.

GDS=Geriatric Depression Scale; SD=standard deviation.

There was no significant difference between GI and GII regarding age, education, sex, living alone, number of medications, use of psychotropic drugs, or level of physical activity, demonstrating homogeneity between groups. There were, however, significant differences for number of comorbidities and depressive symptoms between the participants with and without LBP (Table 1).

The global fall risk for GI and GII was 1.6 and 0.5, which characterized them as having a moderate and mild fall risk, respectively. GI had a significantly higher overall fall risk, greater postural sway and longer reaction time in comparison to GII. In addition, the LBP group had significantly lower quadriceps strength than GII. There was no significant difference for vision and proprioception tests between GI and GII. The elders of GI fell more times in the last 12 months than the elders of GII (Table 2).

**Table 2:** Comparison between groups: fall risk and physiological systems related to the fall risk.

		**Low Back Pain Group (GI)** **n=52**	**Control Group** **(GII)** **n=52**	***p value***	**Mean between-group differences and 95% CI**
		**Mean (SD)**	**Mean (SD)**		
**Global fall risk**		1.6 (0.7)	0.5 (0.8)	0.00^*&^	1.10 (0.72 to 1.48)
**Vision (dB)**		19.5 (1.9)	20.4 (1.3)	0.11^#^	–0.85 (–1.75 to 0.56)
**Proprioception (°)**		3.2 (1.6)	2.5 (1.1)	0.11^#^	0.71 (–0.13 to 1.54)
**Postural Sway (mm)**		154.4 (72.5)	104.6 (33.0)	0.00^*#^	49.78 (13.54 to 86.01)
**Reaction Time (ms)**		334.1 (45.3)	275.2 (52.0)	0.00^*&^	58.95 (33.25 to 84.65)
**Quadriceps Strength (Kg)**		19.1 (8.0)	23.6 (7.1)	0.02^*&^	–4.42 (-8.25 to -0.59)
**Falls 12 months**		2.4 (3.1)	0.4 (0.5)	0.01^*#^	
**% Falls 12 months**		57.3%	34.6%	0.01^*†^	

* Significant difference p<0.01;

^&^ t-test;

# Mann-Whitney U test;

† Chi-square test;

SD=standard deviation; CI=Confidence Intervals for the difference.

Regarding falls, 57.3% of GI fell in the last 12 months, while only 34.6% of the subjects in GII fell in the same period. Among seniors with LBP who fell, 76.6% of falls were accidental and 70% of these occurred outdoors. Four elders were hospitalized, included one for a fractured vertebra. In the GII, 88.8% of falls were accidental and 77.7% occurred outdoors. None of the individuals in this group was hospitalized or had fractures.

Regarding the use of psychotropic drugs, there was no significant difference between the two groups (p=0.052) (Table 1). GI group: antidepressants (fluoxetine) = four individuals; benzodiazepines (mainly clonazepam) = six; barbiturates = one. GII group: antidepressants = one individual; benzodiazepines = two; barbiturates = one.

52% of GI reported having used analgesic or anti-inflammatory treatment for LBP in the last three months and 42% of these said they had taken these drugs in the last 24 hours before the assessment. The most common medications were paracetamol and dipyrone.

## Discussion

The group with LBP presented a higher fall risk, increased postural sway and reaction time, and lower quadriceps strength when compared to the group without LBP. In addition, GI fell more times in the last 12 months in comparison to GII.

In this study, the elders with LBP had a higher overall score in the PPA, indicating an increased fall risk. LBP can change and deteriorate sensory information for postural control originating from the paraspinal muscle. This may be related to an increase in presynaptic inhibition of the muscle afferent due to pain^12^. Acute LBP (ALBP) is perceived as interference, which can lead to an increase in the threshold of nociceptive afferents in the lower back, further causing interference on the spinal motor via and the motor cortex. Therefore, such changes may increase the fall risk in older adults.

LBP can also lead to alterations in the normal upright position, as well as inhibition of muscle activation for the protection of injured tissues^23^. These compensatory changes in posture and muscle activation patterns may occur as a strategy for limiting the spine movements and avoiding movements that trigger pain (kinesiophobia), leading to a change in balance and postural control. Thus, individuals with LBP have higher postural sway and greater displacement of their center of pressure^24^, compared to those without this dysfunction. The factors causing these changes are: limitations in the ability to use the hip strategy due to pain; reduced muscle strength and flexibility of the lumbopelvic region^12^. Moreover, patients with LBP may use postural control strategies that differ from healthy subjects, e.g., greater use of co-contraction of the triceps surae to maintain a standing balance when compared to subjects without LBP^25^. The use of co-contraction of the triceps surae may increase postural sway in these individuals.

The pain associated with LBP can cause an increase in presynaptic inhibition of muscle afferents resulting in prolonged latency due to a decrease in feedback from the muscle spindle^26^. Thus, motor responses are slower, and as evidenced in this study, the GI group had longer reaction time to stimulation as assessed in the PPA.

Another finding in this study was that the elders in GI had lower quadriceps strength than GII. These findings corroborate those of a previous study by Weiner et al.^27^, in which LBP was associated with lower knee extension strength. Muscle weakness may arise from a lack of physical activity; however, the level of physical activity of GI and GII showed no significant difference. Another possible factor that could account for the decrease in muscle strength in the GI group could be the direct effect of pain, referred to as reflex muscle inhibition, which causes prolonged latency in muscle responses^24^. There is loss of protective responses against the threat of falling. In addition, lower knee extensor strength has a significant association with low mobility and increased risk of mortality^28^.

Other results of the present study show that older people with LBP fell more times in the previous year than those in GII. In a previous study of subjects aged ≥49 years, Blyth et al.^29^ found that participants who had moderate to severe pain were more likely to report one or multiple falls in the previous year, when compared to individuals without pain. In another study in older women with a mean age of 65 years, Muraki et al.^30^ found that LBP is independently associated with multiple falls in the previous 12 months.

There was a significant difference between GI and GII for depressive symptoms and comorbidities. The relationship between depression and disability in elders with LBP can be explained by the fact that pain can make these individuals feel helpless and disabled and have less motivation to do their best performance in activities. Symptoms common to depression, such as negative thoughts and self-perceived fatigue, will interfere with how older people with LBP deal with the pain and contribute to the presence of disability^31^. Elders with LBP also had more comorbidities than the ones without LBP, which is supported by Rudy et al.^32^.

Some limitations of the present study should also be considered. The study has a recall bias for falls since the data was collected retrospectively. Measurement of trunk muscle strength in older adults with LBP was not performed; however, this could be a more accurate measure of the influence of LBP on the muscle system. An isokinetic dynamometer could be used. Another study limitation is associated with self-reporting of LBP. Although the survey is limited by the use of self-report to identify LBP^33^, in population cohorts like the BACE, self-reporting of health conditions is an accepted methodology for large surveys when a detailed chart review is not feasible and when concordance between the self-report and medical record review is generally good (κ=0.60). Another limitation is that the sample consists of elders that presented LBP for the first time and chronic patients with an acute episode.

In addition, the study excluded individuals with high-risk factors for falls already established in the literature such as cognitive dysfunctions, disorders of the vestibular system, and neurological diseases. This may have influenced the score for the falls risk and other tests performed in both groups and undermined the extrapolation of results to other individuals and other clinical cases. Nevertheless, these conditions were excluded because they could influence the relationship of LBP with falls. Furthermore, depending on the severity of these symptoms, the individuals might not be able to perform the tests proposed by the PPA. Moreover, these exclusion criteria are common to other studies that used PPA^34^ ^,^ ^35^.

While the causes of falls are complex and multifactorial, the multidimensional evaluation of falls risk by the PPA may be a relevant and advantageous way for clinicians to recognize a set of modifiable risk factors that together explain part of the fall event and, above all, can guide the intervention. The PPA assesses systems already known in the literature as altered by LBP such as proprioception^24^, quadriceps strength^26^, and postural sway^12^ ^,^ ^23^. The PPA uses a function-based, quantitative model and provides a tool for fall risk factor identification and providing direction for intervention.

The PPA is not a commonly available instrument, but physical therapists can access postural control, strength, and reaction time. In addition, the results of this study are particularly relevant for the physical therapists who assist older patients complaining of LBP in an orthopedic setting. Often, these physical therapists are not well trained in geriatrics and gerontology and may not even ask their patients about falls. Therefore, they should be aware of an increased risk of falling among these patients.

## Conclusion

The results of this study indicate that older people with LBP have greater risk for falls than those without LBP, and physical therapists in the clinical setting should be aware of an increased risk of falling among their patients. Thus, we conclude that fall risk screening may be sensible in elders with LBP.
